# Review of Liquid Metal Fiber Based Biosensors and Bioelectronics

**DOI:** 10.3390/bios14100490

**Published:** 2024-10-09

**Authors:** Xiaotong Liu, Hui Xu, Jiameng Li, Yanqing Liu, Haojun Fan

**Affiliations:** Institute of Disaster and Emergency Medicine, Tianjin University, Tianjin 300072, China; liuxiaotong_11@tju.edu.cn (X.L.); jiameng@tju.edu.cn (J.L.)

**Keywords:** liquid metals, conductive fiber, biosensors, bioelectronics

## Abstract

Liquid metal, as a novel material, has become ideal for the fabrication of flexible conductive fibers and has shown great potential in the field of biomedical sensing. This paper presents a comprehensive review of the unique properties of liquid metals such as gallium-based alloys, including their excellent electrical conductivity, mobility, and biocompatibility. These properties make liquid metals ideal for the fabrication of flexible and malleable biosensors. The article explores common preparation methods for liquid metal conductive fibers, such as internal liquid metal filling, surface printing with liquid metal, and liquid metal coating techniques, and their applications in health monitoring, neural interfaces, and wearable devices. By summarizing and analyzing the current research, this paper aims to reveal the current status and challenges of liquid metal conductive fibers in the field of biosensors and to look forward to their development in the future, which will provide valuable references and insights for researchers in the field of biomedical engineering.

## 1. Introduction

With the rapid development of biotechnology and materials science, biosensing technology has become a core component of modern biomedical engineering. Especially in the design and functional realization of biosensors, the application of new materials continues to drive the progress in this field. In recent years, conductive fibers, as an important research direction, have gradually been recognized as a focus of researchers’ attention for their potential applications in the fields of biosensing [1,2,3], human-computer interaction [4,5], soft robotics [6,7], and flexible energy [8]. For example, in the field of wearable devices, conductive fibers can be woven into fabrics to provide not only a comfortable fit but also stable monitoring of physiological signals [9,10,11,12].

Conductive fibers are usually prepared from materials with good electrical conductivity, which can be metals, semiconductors, or conductive polymers. Conventional solid metallic materials, such as gold, silver, copper, and platinum, are widely used due to their excellent electrical conductivity [13,14,15,16,17]. However, these materials are typically hard and lack flexibility, limiting their use in certain applications where high performance and flexibility are required. To overcome these limitations, researchers have developed fibers based on conductive polymers [18,19], carbon–based materials [20,21], and nanocomposites [22,23,24,25], which exhibit good electrical conductivity and higher flexibility.

Liquid metal differs from conventional solid metals in that they assume a liquid state at or near room temperature. This unique physical state endows them with excellent electrical conductivity [26,27], low melting point phase transition [28], good flowability [29,30], adhesion [31], and very high biosafety [32]. These properties make liquid metals ideal for the fabrication of flexible conductive fibers, which are particularly suitable for applications requiring high flexibility and stretchability, such as in the field of biomedical sensors. Common manufacturing methods for liquid metal-based conductive fibers include internal filling with liquid metal [33], surface printing with liquid metal [34], and liquid metal coating [35]. These conductive fibers have a wide range of applications involving wearable devices, real-time health monitoring, environmental monitoring, implantable medical devices, and many others [36].

**Figure 1:**
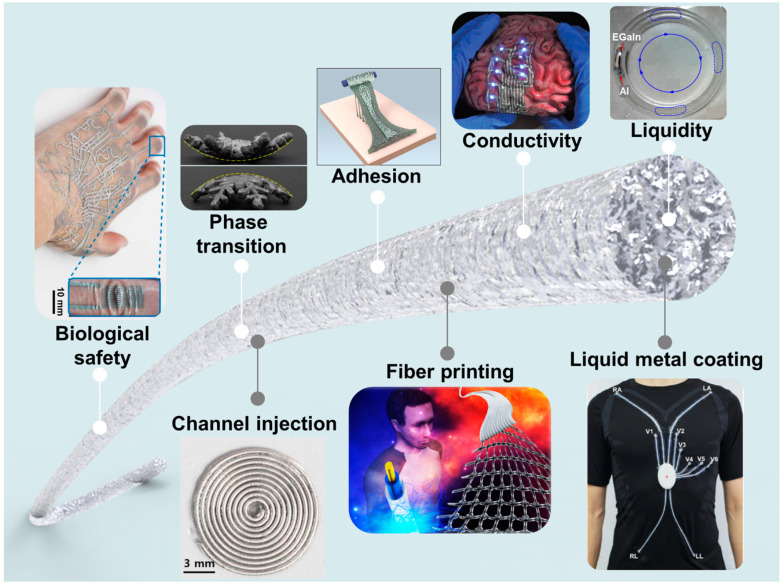
Unique properties of liquid metals and their realization as liquid metal conductive fibers.

The main purpose of this paper is to discuss the current status and development potential of liquid metal conductive fibers in the field of biosensors by summarizing and analyzing the existing literature. The article first introduces the properties and research progress of liquid metals, then elaborates on the common fabrication methods of liquid metal-based conductive fibers and analyzes the practical cases of these fibers in different biosensing applications, as shown in Figure 1. In addition, this paper will discuss the problems and challenges in the current research and suggest possible directions for future research. The aim of this paper is to promote the further application and development of liquid metal-based conductive fibers in the biomedical field and to provide new ideas and inspirations for future scientific research, which will, in turn, promote the innovation and advancement of biosensor technology.

## 2. The Unique Properties of Liquid Metals

Unlike the traditional rigid conductive materials, liquid metals, like gallium and gallium-based alloys, have gradually attracted the attention of researchers for their good electrical and thermal conductivity, liquidity, solid-liquid transition, and excellent biosafety. They have more potential application scenarios in the field of manufacturing smart fibers compared with traditional metals. In this section, some of the unique properties of gallium-based liquid metals that facilitate the fabrication of liquid metal fibers will be discussed.

### 2.1. High Electrical Conductivity

**Figure 2:**
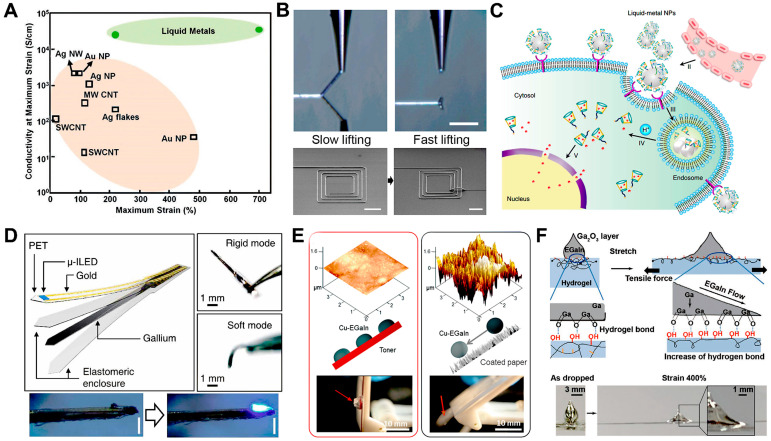
The unique properties of liquid metals. (**A**) Comparison of conductivity at maximum strain of various stretchable conductors [37]. Copyright: (2018) WILEY-VCH. (**B**) Photograph of lift-off and cutoff of liquid metals from the substrate; SEM images of reconfigured square coils [39]. Copyright: (2019) AAAS. (**C**) The pH-responsive delivery of Dox by liquid metal/Dox-L nanocomposites to the nuclei for the targeted cancer therapy [42]. Copyright: (2015) Springer Nature. (**D**) Conceptual illustration of the fabrication scheme and photographs of the neural probe with adjustable stiffness in rigid and soft modes [48]. Copyright: (2019) AAAS. (**E**) Scanning probe microscopy (SPM) morphology characterization, schematic illustration, and inclining experiment with the liquid metals on toner substrate and coated-paper substrate [52]. Copyright: (2020) American Chemical Society. (**F**) Schematic illustrations and photographs of the surface creation of liquid metals on hydrogel when stretched to a strain of 400% [54]. Copyright: (2020) WILEY-VCH.

In general, the electrical conductivity of metallic materials is much higher than that of non-metallic materials, as shown in Table 1. Figure 2A demonstrates the high conductivity of liquid metals compared to other commonly used flexible conducting materials at maximum strain. For example, the conductivity of gallium-indium alloys (EGaIn: 3.4 × 10^6^ S m^−1^), which are commonly used to prepare flexible circuits, is much higher than that of other non-metallic materials (Carbon: 1.8 × 10^3^ S m^−1^, CNT: 5.03 × 10^3^ S m^−1^, PEDOT. PSS: 8.25 × 10^3^ S m^−1^) [37,38]. Therefore, making liquid metals as conductive coatings for fibers can significantly improve their conductivity and ensure the proper functioning of the corresponding electronic circuits.

### 2.2. Fluidity

At room temperature, liquid metals can remain liquid with excellent fluidity, which allows them to follow the deformation of fibers without limiting their deformability. In addition, the injection of liquid metals into microfluidic structures has been widely used to pattern and package highly deformable and reconfigurable electronic devices, sensors, antennas, and interconnects. For example, the high fluidity of the liquid metals allows them to be infused into microfluidic channels and to be extruded from the microfluidic channels or syringes to be printed on flexible substrates for the fabrication of a variety of flexible electronic circuits, as shown in Figure 2B [39]. In addition, Majidi et al. have infused liquid metals with high melting points into molds made of flexible materials for the rapid fabrication of three-dimensional electronic devices with complex structures [40].

### 2.3. Biosafety

Gallium-based liquid metals have favorable biosafety. Due to their low vapor pressure and limited solubility in water, gallium-based liquid metals are essentially inaccessible to the human body, and their cytotoxicity is much lower than that of the highly toxic metal mercury. Previous research has shown that the concentration of gallium ions released from aqueous solutions of gallium-indium alloys after prolonged immersion is well below safety standards. Experiments in which indium gallium alloy was co-cultured with biological cells showed that the cell survival rate was close to 100%. In addition, there were no significant toxic side effects when gallium-indium alloy was injected subcutaneously into mice [41]. Additionally, Lu et al. prepared deformable core-shell nanospheres composed of liquid metals, demonstrating the ability to fuse and subsequently degrade under weakly acidic conditions, which promote Dox release from acidic endosomes following cellular internalization, as shown in Figure 2C. Equipped with hyaluronic acid, a tumor-targeting ligand, the preparation showed enhanced chemotherapy inhibition in xenografted homozygous mice. These liquid metal core-shell nanospheres with fusible and degradable behavior under physiological conditions provide a new strategy for engineering therapeutic diagnostic agents with low toxicity [42]. In addition, an injectable bone cement material has been prepared using a bismuth-indium-tin-zinc (Bi/In/Sn/Zn) alloy with bismuth metal as the main body. This liquid metal bone cement not only has high mechanical strength but also only needs to be heated to make it melt. Therefore, the repair of a specific location of the bone can be accomplished by injection, and when the bone grows and recovers, it is also possible to pump the liquid metal out of the body by injection, avoiding the harm to the human body caused by the secondary surgery [43]. In addition, due to the high imaging ability of liquid metals in the CT image, the melting point of 29.8 °C gallium metal was injected into the isolated heart, kidneys, and other organs rich in blood vessels, making the organ in the capillary in the CT image show a very good imaging effect. This technique enables the visualization of capillary distribution in isolated tissues. In summary, the biosafety of liquid metals allows the fabrication of conductive fibers that can be used in a wide range of biomedical fields.

### 2.4. Solid-Liquid Phase Transition

Due to their low melting points, liquid metals can respond to changes in external temperature at room temperature, causing them to transition between solid and liquid states. This phase change property of liquid metals allows for significant changes in thermal conductivity, hardness, shape, and volume and can be used to fabricate 3D circuits with adjustable stiffness. For example, Liu et al. utilized 3D printing methods to print liquid metals with high melting points into a variety of 3D structures, including liquid metal balls, liquid metal rods, the frustum of a cone structure, and a cylinder structure [44]. In addition, liquid metals have been crushed and prepared into micro and nanoparticles, and the morphological variation caused by the phase change in liquid metals has been exploited to produce spikes that can destroy the surrounding tumor cells and provide a specific therapeutic effect on the diseased biological tissues [45,46,47]. Sun et al. conducted biocompatibility tests for over 120 days, demonstrating the favorable long-term biocompatibility of liquid metal. Also, there have been studies in which liquid metal has been infused into microtubulars to make needle-like electrodes. Liquid metals are used as an innovative material to regulate the mechanical strength of the electrodes, and the needle electrodes have high mechanical strength and can be easily inserted into the neural tissues in a low-temperature environment. On the contrary, after the electrode is implanted into the brain tissue, the temperature is raised to fuse the liquid metals. Thus, the electrode is in a state of lower mechanical strength, which significantly improves the compliance of the electrode to alleviate the mechanical mismatch between the tissue and the electrode and improves the long-term implantation stability of the electrode, as shown in Figure 2D [48]. The unique advantages of such electrodes offer a wide range of application scenarios in tumor therapy and long-term monitoring, as well as stimulation and irritation of the nervous system. Thus, the solid-liquid phase change properties of liquid metals can be utilized to fabricate smart fibers with phase change functionality and be used for implantable electronics and three-dimensional electronic circuit fabrication.

### 2.5. Interface Adhesion

Liquid metals have high surface tension, which makes them difficult to adhere to the surface of materials with high roughness, such as skin, fabric, paper, and 3D-printed models [49,50,51]. To solve this problem, some studies have proposed schemes to improve the adhesion of liquid metals by pre-covering the rough substrate surface with an adhesive coating to reduce the substrate roughness. For example, by pre-printing pro-liquid-metal toner on a rough substrate, Guo et al. selectively adhered liquid metal to a designed pattern and transferred metal circuits to an uneven and deformable Ecoflex surface to form 3D circuits. In addition, the method can combine the laser printer with thermal transfer technology to enable liquid metal circuit fabrication on a variety of substrates, saving the cost and time of preparation and advancing next-generation flexible electronics manufacturing, as shown in Figure 2E [35,52]. 

Previous studies have found that liquid metals are easily oxidized in air, and their surfaces are covered with an oxide film. The presence of the oxide film not only reduces the surface tension of the liquid metals but also makes them have high adhesion on the surface of a variety of polymer materials, realizing their stable adhesion on the substrates [53]. For example, Park et al. found that liquid metals can adhere to aliphatic alkyl-chain hydrogel substrates with many hydroxyl groups on the surface, and complex flexible circuits can be formed by combining with stencil printing methods, as shown in Figure 2F [54]. More importantly, liquid metals on the hydrogel can automatically adjust their surface to form a strong interfacial adhesion during mechanical deformation of the substrate. This results in the fabrication of liquid metal electrodes with reversible stretching, self-repairing, and water swelling capabilities and very high conductivity and deformation. Therefore, this interfacial adhesion property of liquid metals can realize its conformal adhesion on the surface of polymer fibers and can maintain stable electrical conductivity under the substantial deformation state of fibers.

## 3. Three Kinds of Liquid Metal Fibers

The exceptional stretchability and electrical conductivity of liquid metals render them particularly well–suited for the fabrication of stretchable conductive fibers. In recent years, numerous preparation strategies have been developed to integrate an elastic fiber matrix with a liquid metal conductive filler. These preparation methods can be categorized based on the manner in which the liquid metal is incorporated into the elastic fiber matrix. The primary classifications include internally filled liquid metal-based tubular textile fibers, surface-printed liquid metal-based fibers, and liquid metal-coated fibers, as shown in Table 2.

## 4. Internally Filled Liquid Metal-Based Tubular Textile Fibers

### 4.1. Preparations

**Figure 3:**
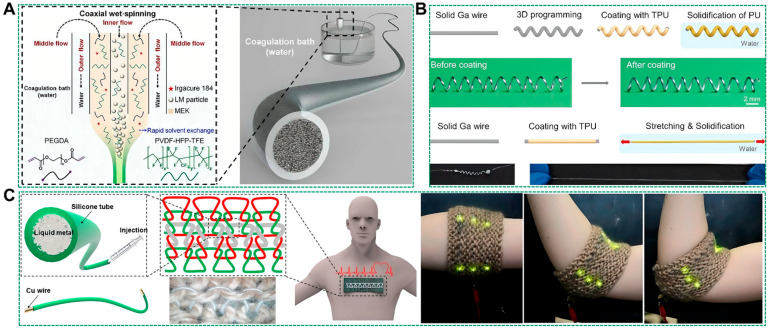

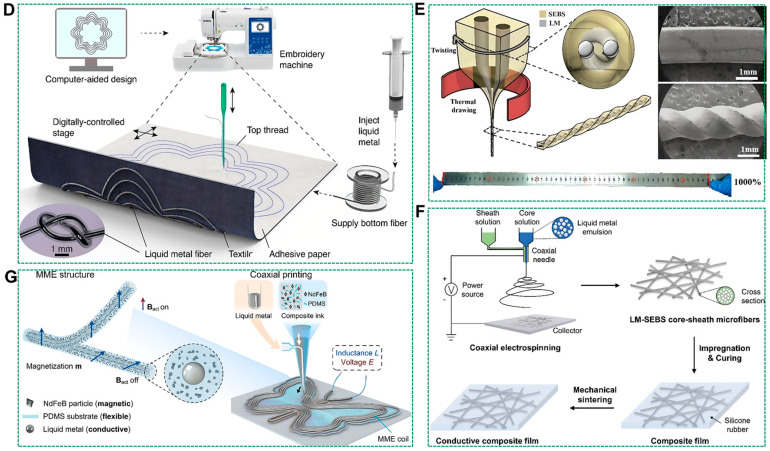
Preparation methods of internally filled liquid metal-based tubular textile fibers. (**A**) Schematic setup and the main components for the coaxial wet-spinning process for producing liquid metal sheath-core microfibers [55]. Copyright: (2021) AAAS. (**B**) Schematic illustration of the preparation of shape-programmable liquid metal fibers and the reduction in the fiber diameter during PU solidification [63]. Copyright: (2022) MDPI. (**C**) Preparation, structure, and application of liquid metal fibers with a knitted structure [65]. Copyright: (2023) MDPI. (**D**) Illustration of the digital embroidery process. Liquid metal fibers consist of perfluoroalkoxy alkane tubing infiltrated with Galinstan [66]. Copyright: (2022) Springer Nature. (**E**) Schematic presentation of the manufacturing process of the helical two liquid metal channel SEBS fibers. SEM image of the external surface of unhelical two liquid metal channel SEBS fibers with a twist angle of 45° [67]. Copyright: (2024) Elsevier. (**F**) Fabrication procedure of liquid metal fiber network composites [69]. Copyright: (2024) WILEY-VCH. (**G**) Design and functional composition of the hybrid magnetic-mechanical-electrical structure [70]. Copyright: (2023) Springer Nature.

The incorporation of liquid metals into the interior of tubular elastic fibers is a crucial step in the preparation process. However, the excellent fluidity of liquid metals encapsulated in pipes makes them prone to leakage. Additionally, the high surface tension of liquid metals can lead to separation from the tube during large-scale tensile deformation, resulting in wire breakage. To address these challenges, Zheng et al. introduced a coaxial wet-spinning process for continuously producing intrinsically stretchable and highly conductive yet stable liquid metal sheath-core microfibers [55]. The sheath of these microfibers consists of a double-network fluoroelastomer with good elasticity, while the core is composed of a composite material consisting of fluoroelastomer and percolated liquid metal nanoparticles. The sheath-core structure and dipole-dipole interactions between the fluoroelastomer and the liquid metals enable embedded particles in the core to conformally deform upon stretching (Figure 3A). These microfibers can be stretched up to 1170%, achieving very high conductivity (4.35 × 10^4^ S m^−1^) and minimal resistance change (only 4% at 200% strain) when fully activating their conductive path. Importantly, embedding liquid metal particles within fluoroelastomer effectively prevents leakage. However, it should be noted that conductive complexes fabricated by mixing liquid metal particles with polymers exhibit significantly lower conductivity compared to liquid metal monomers [56]. Therefore, most of the current studies have used the strategy of injecting liquid metal directly into the interior of hollow elastic tubular fibers to fabricate liquid metal conductive fibers [57,58,59,60,61,62]. This simple and convenient method can be used to fabricate ultra-long liquid metal fibers in large quantities.

In order to fabricate circuits with specific functions using liquid metal conductive fibers, it is necessary to assemble the conductive fibers into specific 2D or 3D structures. For instance, Ma et al. have developed a method for creating shape-programmable liquid metal fiber [63]. As depicted in Figure 3B, this approach leverages the plasticity of gallium [64], a solid-state material capable of shaping 1D metal wires into intricate 3D helical structures. Following the application of a thin layer of polyurethane (PU) elastomer onto the fibers, the resulting 3D structure can be permanently preserved even as the gallium transitions into its liquid state. These 3D liquid metal fibers exhibit an enhanced stretchability of up to 1273% and demonstrate consistent conductance over strains reaching up to 283%. Furthermore, by stretching the fibers during the PU coating process, their diameter can be reduced from 343 µm to 108 µm. This presents an accessible means for producing lightweight and ultrafine liquid metal fibers.

In addition, liquid metal fibers can be integrated with established garment manufacturing processes to facilitate the rapid and automated production of fiber structures. For instance, Ping et al. fabricated a range of circuits using a knitting technique that incorporated liquid metal conductive fibers with cotton threads. The knitted structure of these fibers notably diminishes the variation in electrical resistance during fabric deformation. The liquid metal fiber is fabricated by injecting liquid metals into a silicone tube using a syringe, with copper wires inserted at both ends of the tube to prevent leakage and facilitate connections to other circuits, as depicted in Figure 3C [65]. Lin et al. created liquid metal fibers by infusing liquid metals into perfluoroalkoxy (PFA) tubing and affixed them to garments using a digital embroidery method, as illustrated in Figure 3D [66]. This method is scalable and yields fibers with superior electrical and mechanical properties. Their compatibility with the digital embroidery process allows for the application of established computer-aided design technologies in textile manufacturing. These liquid metal textiles exhibit durability against repeated stress and strain and are resistant to wear after multiple washing and drying cycles.

Furthermore, the direct fabrication of liquid metal fibers into specific 3D structures is a widely adopted strategy in the liquid metal fiber preparation process. For instance, Zhang et al. employed a one-step thermal drawing and spiraling technique to heat and elongate a poly styrene-b-(ethylene-co-butylene)-b-styrene (SEBS) preformed rod, simultaneously shaping it into helical structures and continuously collecting it with an electrical motor. Subsequently, liquid metals were injected into the dual channels of the helical fibers, yielding superelastic thermally drawn liquid metal fibers with a helical architecture, as illustrated in Figure 3E [67]. These fibers exhibit a core-shell structure, with the core containing two helical liquid metal channels and the shell consisting of superelastic SEBS. Such helically structured liquid metal fibers are amenable to mass production for applications in human motion monitoring, energy harvesting, and thermal management [68]. Ma et al. utilized liquid metal/SEBS core-sheath microfibers with diameters ranging from 10 to 20 μm to create a disordered network of conductive fibers. The interconnected liquid metal fibers serve as conductive pathways, enabling the realization of liquid metal composites with low density and high conductivity, suitable for large-scale, cost-effective, lightweight, and sustainable applications, as depicted in Figure 3F [69]. Three-dimensional printing technology, with its ease of operation and flexibility, offers significant advantages for patterning liquid metal fibers. For example, Zhang et al. applied a one-step coaxial printing method to fabricate a hybrid magnetic-mechanical-electrical core-sheath fiber, as shown in Figure 3G [70]. This fiber comprises a liquid metal core and a soft magnetoactive composite sheath with programmable magnetic responsiveness, high electrical conductivity (2.07 × 10^6^ S m^−1^), robust mechanical properties (150% elongation and 0.87 MPa), and functional durability within a single structure. Leveraging digitally controlled coaxial printing, complex 2D/3D coils with hybrid magnetoactive and electrically conductive properties can be produced in a single step.

### 4.2. Applications

**Figure 4:**
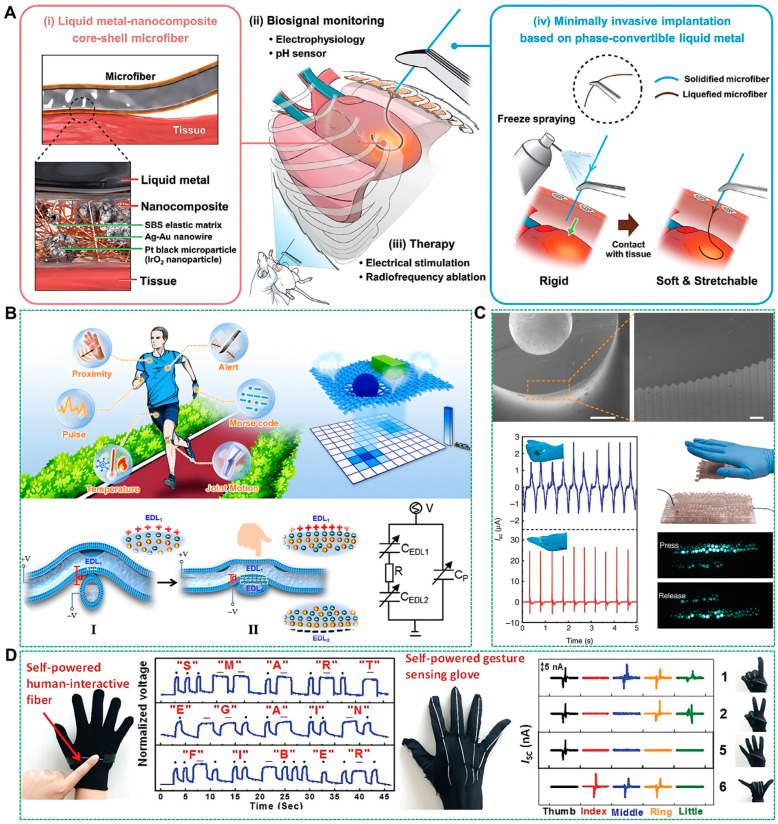
Application of tubular textile fibers internally filled with liquid metal. (**A**) Schematic illustration of the microfiber structure and its minimally invasive administration protocol. (i) The microfiber composed of a liquid metal core and nanocomposite shell is conformally applied to the tissue surface and performed. (ii) biosignal monitoring and (iii) electroceutical therapies. (iv) The solidified microfiber can penetrate tissues, and it recovers its original softness in response to the body temperature after implantation [74]. Copyright: (2024) WILEY-VCH. (**B**) Schematic diagrams showing the working mechanism and the versatile potentials of the fiber-based capacitive sensor acting as a wearable pressure sensor or temperature sensor [80]. Copyright: (2023) American Chemical Society. (**C**) SEM image of a fiber with the microtextured surface; waveforms of the textile triggered by mechanical stimuli: hand grasping (upper part) and hand tapping (bottom part); demonstration of 100 LEDs lighted up by the textile under-tapping [81]. Copyright: (2020) Springer Nature. (**D**) Left: Demonstration of ISLMF as a self-powered human-device interface. Right: Demonstration of ISLMF as a self-powered gesture-sensing glove [82]. Copyright: (2021) WILEY-VCH.

Internally filled liquid metal-based tubular textile fibers, characterized by their liquid metal core, exhibit high electrical conductivity [71], high compliance [72], and undergo a solid-liquid phase transition [73], endowing them with unique potentials across various applications. In biomedical engineering, these fibers, due to their exceptional biocompatibility and electrical conductivity, are poised to revolutionize the development of neurostimulation devices. Moreover, they have demonstrated potential in smart textiles, pressure sensing, and energy storage systems. For instance, implantable electrical stimulators are crucial for the treatment of non-pharmacological diseases. However, traditional rigid implants often encounter issues such as reduced signal-to-noise ratio, stimulation fatigue, scar formation, and inflammatory responses due to mechanical mismatch at the device-tissue interface. To facilitate the minimally invasive implantation of flexible stimulators, Kim et al. developed needle-like, minimally invasive, implantable multifunctional biphasic microfibers leveraging the solid-liquid phase change properties of liquid metals for independent implantation and flexible conformation, as depicted in Figure 4A [74]. The microfibers featured a phase-convertible liquid metal core encased in a nanocomposite shell composed of a blend of gold-coated silver nanowires, elastic block copolymers, and functional additive particles. Before implantation, the core is solidified via freeze-spraying to enhance stiffness for tissue penetration. Post-implantation, the core liquefies at body temperature, restoring the microfibers’ inherent flexibility for a seamless tissue fit. The core-shell configuration endows the microfibers with the combined benefits of liquid metal and nanocomposites, yielding high stretchability, conductivity, and strain insensitivity. These multifunctional microfibers are suitable for a variety of tissue surfaces, including the stomach, muscle, and heart, presenting opportunities for electrophysiological recordings, pH sensing, electrical stimulation, and radiofrequency ablation. In parallel, gallium, with its phase transition capability, low glass transition temperature, and biocompatibility, is an ideal candidate for crafting variable stiffness medical needles. Addressing the challenge of tissue damage and the risk of blood-borne pathogen transmission due to the high stiffness of intravenous needles, Jeong et al. introduced a variable-stiffness intravenous needle with a gallium frame structured in two U-channels [75]. The outer U-channel frame encloses the inner U-channel platform, creating a closed rectangular hollow structure, subsequently encapsulated with silicone renowned for its superior tear strength.

Additionally, the liquid metals within fiber tubes altered their shape under external pressure, thereby inducing electrical changes in the fibers [76,77]. Capitalizing on this property, liquid metal fibers have been integrated into various structures for pressure detection, facilitating the creation of smart garments [78,79]. For instance, Chen et al. developed an innovative fiber-based iontronic sensor with superior pressure and temperature sensing capabilities. This sensor was constructed by combining two intersecting hollow and porous ionogel fibers filled with liquid metals, as illustrated in Figure 4B [80]. As a pressure sensor, it boasts a high detection resolution of 1.16 Pa, a high sensitivity of 13.30 kPa^−1^, and a broad detection range of approximately 207 kPa. As a temperature sensor, it exhibits high-temperature sensitivity of 25.99% °C^−1^, high resolution of 0.02 °C, and demonstrates excellent repeatability and reliability. Leveraging these exceptional sensing attributes, the fabricated sensor can detect a spectrum of pressure signals, from subtle pulses to significant joint movements, as well as the proximity of various objects. Moreover, a large-area fiber array can be effortlessly woven to produce a pressure map, enabling the visual differentiation of the loaded object’s position, dimensions, and form. This research presents a universal strategy for the design of fiber-shaped iontronic sensors for wearable electronics.

A critical component of biosensors is a sustained and efficient energy supply system. Traditional battery devices, with their bulky size and frequent recharging requirements, have inherent limitations. Liquid metal fibers offer a promising solution to these challenges. For example, Dong et al. fabricated triboelectric fibers with intricate microstructures using a sophisticated multimaterial thermal drawing technique. These fibers’ performance rivals that of state-of-the-art triboelectric nanogenerators, heralding new opportunities for the evolution of advanced functional fibers and fabrics [81]. Given that commonly utilized materials in everyday life tend to exhibit positive electrode properties, the research team employed Geniomer, known for its excellent flexibility and robust negative electrode characteristics, as the sheath. The liquid metal Galinstan was used as the electrode to assemble a super-stretchable triboelectric fiber capable of withstanding elongation of up to 560% without breaking. Additionally, by designing periodic square-shaped patterns on the preform and a multi-electrode surface structure, the fiber’s performance was significantly enhanced. When integrated into functional fabrics, the open-circuit voltage and short-circuit current can peak at 490 V and 175 nC, respectively. This triboelectric fiber is capable of harnessing the energy generated by human movement. For example, the grasping action of a textile can yield substantial open-circuit voltage (Voc), short-circuit current (Isc), and short-circuit transferred charge, reaching approximately 260 V, 2.5 μA, and 85 nC, respectively. Such levels of output are adequate to power 100 light-emitting diodes (LEDs), as depicted in Figure 4C. Furthermore, liquid metal fibers can be crafted into self-powered strain sensors for human-computer interaction. Dickey et al. introduced intrinsically stretchable liquid-metal fibers (ISLMF) that can capture mechanical energy from human motion and electromagnetic energy from nearby electrical devices [82]. These fibers can be directly sewn onto the index finger area of a glove, converting finger touches into actionable information. The Voc produced by static or transient touches can be recognized as “dash” or “dot” signals in Morse code sequences for information transmission. Figure 4D (left) displays a series of Voc signals generated by a finger touch, spelling out the phrase “SMART AGAIN FIBER.” The fiber can also detect finger-bending angles. Figure 4D (right) illustrates the real-time Voc and Isc generated in response to various gestures.

## 5. Surface-Printed Liquid Metal-Based Fibers

### 5.1. Preparations

**Figure 5:**
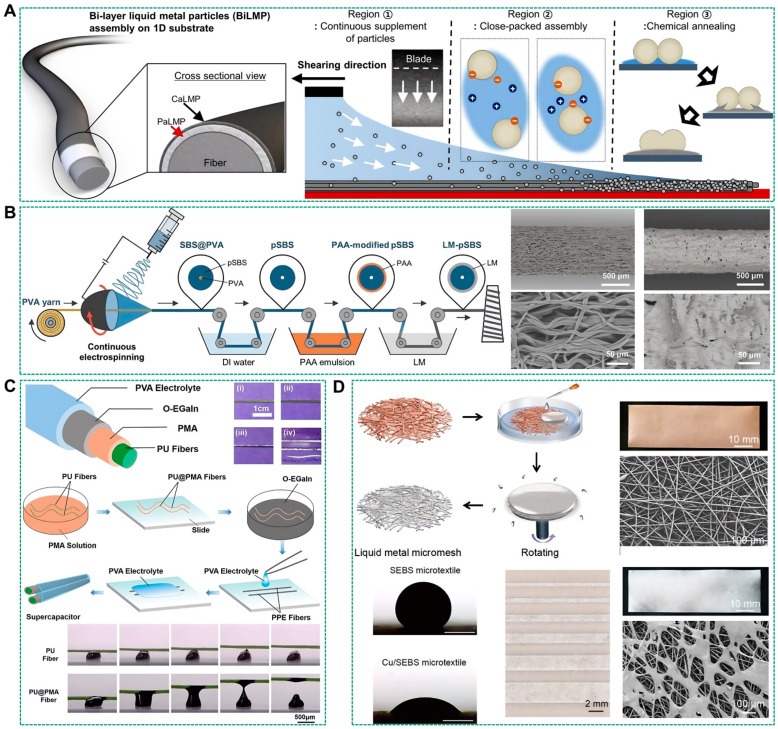
Preparation of the surface-printed liquid metal-based fibers. (**A**) Schematic illustration of BiLMP-coated fiber [84]. Copyright: (2023) Springer Nature. (**B**) Schematic illustration and SEM images of the fabrication of LM-pSBS fiber with one layer of LM coated on the surface of pSBS fiber [85]. Copyright: (2024) WILEY-VCH. (**C**) Schematic structure and the fabrication process of PU@PMA@EGaIn (PPE) fibers. Photos of the fibers and the supercapacitor [86]. Copyright: (2021) American Chemical Society. (**D**) Schematic illustration of the fabrication process and optical and SEM images for the liquid metal micromesh on the SEBS microfiber textile. Contact-angle images of liquid metal droplets on the SEBS microtextile and Cu-coated SEBS microtextile after treatment in NaOH aqueous solution [87]. Copyright: (2022) American Chemical Society.

Different from the liquid metal stretched conductive fibers prepared by the traditional pipe infusion method, the conductive fibers with liquid metal printed on the surface use the liquid metals as the conductive coating, which are in the outermost layer of the smart fibers. Exposing the liquid metal coating in the outermost layer can make full use of the low melting point and contact wetting characteristics of liquid metals to achieve the regulation of mechanical properties of smart fibers in the solid-liquid conversion state, as well as the regulation of electrical properties of multiple groups of fibers with liquid-phase welding enablement. Furthermore, leveraging the weaveability of smart fibers, multifunctional liquid metal conductive fiber fabrics can be engineered, underscoring their potential in wearable electronics [83]. For instance, Lee et al. have created a highly durable and stretchable conductive fiber, termed BiLMPs, depicted in Figure 5A [84]. The fabrication process involves a two-stage suspension shearing-based coating method. Initially, liquid metal particles (PaLMPs) are affixed to the polymer fiber’s surface to create a conductive coating. In the subsequent stage, the top layer is coated with carbon nanotube-attached liquid metal particles (CaLMPs), enhancing mechanical durability and initial conductivity. 

The liquid metal coating on the fiber surface significantly impedes the exchange of gases or liquids between the external environment and the fiber interior, leading to a substantial waste of the fiber’s internal volume due to its nonpermeability. To mitigate this issue, Li et al. developed porous and highly SBS fibers through a continuous electrospinning process onto a commercial water-soluble polyvinyl alcohol (PVA) yarn [85]. During electrospinning, SBS was attracted to the PVA surface by electrostatic forces, creating SBS@PVA fibers with a core-shell structure. The PVA core was subsequently dissolved using deionized water, yielding porous and elastic SBS fibers (pSBS). The pSBS fibers were then soaked in a polyacrylic acid (PAA) emulsion, which prevented liquid metal agglomeration by forming hydrogen bonds with the oxidized liquid metal through carboxylic acid groups (–COOH), thereby enhancing the superhydrophilicity of the pSBS fibers. This treatment reduced the contact angle of the liquid metal on pSBS from 137.2° to 18.5°. The final liquid metal-pSBS composite fiber was achieved by applying liquid metals to the surface of the PAA-modified pSBS fiber (Figure 5B). Leveraging the additive manufacturing capabilities of electrospinning and encapsulation of liquid metal-pSBS fibers is straightforward. In a typical process, liquid metal-pSBS fibers serve as a collection substrate for electrospinning, with newly electrospun SBS fibers preferentially covering the liquid metal-pSBS fibers due to electrostatic attraction, thus encapsulating them. This encapsulation provides a protective layer that maintains the fibers’ electromechanical properties under various deformations. The interconnected porous structure of liquid metal-pSBS fibers ensures high breathability and permeability to air, moisture, and liquids. Alongside other cutting-edge stretchable conductive fibers, liquid metal-pSBS offers a material strategy that achieves ultrahigh conductivity, stretchability, and permeability and is amenable to continuous and multilayer manufacturing processes.

Furthermore, in order to solve the problem that gallium-indium alloy has high mobility and is difficult to stably adhere to the fiber surface, Guo et al. improved the preparation process of liquid metal smart fibers by using semiliquid metal material (Cu-EGaIn) with a strong shaping ability as the fiber conductive coating [34]. This Cu-EGaIn doped with solid metal particles can not only maintain the original adhesion state stably for a certain period but also retain the characteristics of liquid metals with low melting points and contact wetting. In order to improve the adhesion of the Cu-EGaIn on the fiber surface, this study pre-coated a layer of polymethacrylate (PMA) coating on the fiber surface to provide high adhesion to Cu-EGaIn. Inspired by this, Duan et al. applied eutectic gallium-indium alloy mixed with metal oxide microparticles (O-EGaIn) on the surface of PU fibers pre-attached with PMA coating and prepared PU@PMA@EGaIn (PPE) fibers, as shown in Figure 5C [86]. Finally, two liquid metal conductive fibers and metal oxide particles were combined to prepare fully flexible fiber supercapacitors with high flexibility and high capacitance. To obtain higher capacitance, metal oxides, including MnO_2_ and Fe_3_O_4_ particles, were introduced. The results show that both MnO_2_ and Fe_3_O_4_ particles can significantly increase the capacitance.

Despite the high deformability inherent in gallium-based liquid metals, the woven mesh structures of liquid metal fibers often result in a significant barrier to gas and liquid permeation. This limitation can impede the functionality of the fibers, especially in sensor applications where internal volume utilization is crucial. To counteract this, Ma et al. have introduced a novel liquid metal micromesh integrated with electrospun microfiber textiles, resulting in a highly permeable and ultra-stretchable conductor. The fabrication process involves dropcasting liquid metals onto the elastomeric microfiber textile, followed by high-speed rotation to eliminate excess coating. This liquid metal micromesh exhibits low sheet resistance, ultrahigh stretchability exceeding 1000% strain, and enhanced mechanical durability, as illustrated in Figure 5D [87]. The porous structure of the micromesh ensures high steam permeability and wearer comfort, comparable to conventional textiles. Moreover, the micromesh’s conformal interface with the skin results in lower contact impedance than state-of-the-art Ag/AgCl gel electrodes, underscoring its potential for use in multifunctional electronic systems and stretching the horizons for applications in the realm of wearable electronics.

### 5.2. Applications

**Figure 6:**
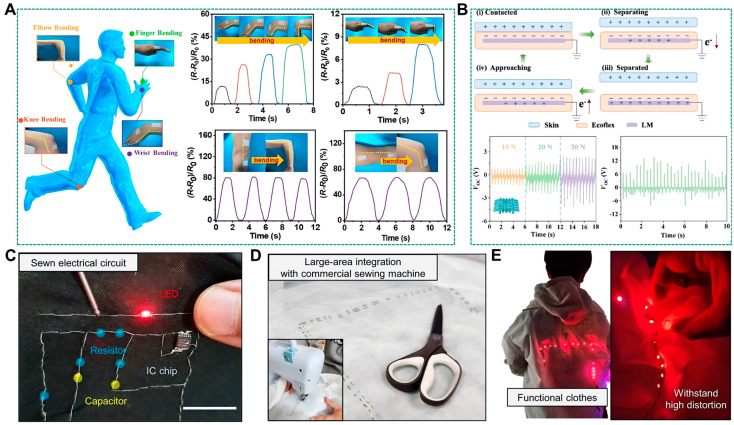
Application of the surface-printed liquid metal-based fibers. (**A**) PPE fibers are used as bending sensors in detecting human motions [89]. Copyright: (2020) American Chemical Society. (**B**) Schematic diagram of single electrode power generation mechanism based on a conductive protein fabric TENG and output voltage of single layer fabric under different loads [90]. Copyright: (2024) American Chemical Society. (**C**) Image of a sewn electrical circuit with a BiLMP-coated fiber [84]. Copyright: (2023) Springer Nature. (**D**) Image of large-area integration of BiLMP-coated fibers on commercial cloth [84]. Copyright: (2023) Springer Nature. (**E**) Image of BiLMP fiber-integrated smart clothes [84]. Copyright: (2023) Springer Nature.

Conductive fibers featuring liquid metals printed on their surface have garnered significant interest for their broad applicability in wearable electronics, body electrodes, and flexible sensors. This interest stems from their straightforward fabrication process, ease of integration, and distinctive benefits, such as the capability to be integrated into flexible textiles or affixed directly onto the skin [88]. For instance, Chen et al. demonstrated that by wrapping liquid metals around polymer fibers. It is possible to confer upon these fibers metallic-like electrical conductivity. Remarkably, the fibers maintain a conductivity level on the order of 10^5^ S m^−1^, even under substantial stretching up to 500% [89]. These fibers excel not only as high-performance flexible conductors but also serve as fiber-type stretch sensors due to their stable and consistent operational characteristics. Such sensors applied to the human body can accurately detect the bending angles of various joints, as illustrated in Figure 6A. Yuan et al. explored the power generation capabilities of liquid metal-infused flexible fabrics [90], focusing on the single-electrode triboelectric nanogenerator (TENG), as shown in Figure 6B. The TENG operates on the principle of electron transfer between the skin and the fabric. Upon contact, electrons move from the skin to the fabric surface, creating a positive charge on the skin and a negative charge on the fabric (i). As the skin separates from the fabric, the resulting electrostatic potential drives electrons from the TENG to the ground, producing a constant electric current (ii) and (iii). Upon recontact, electrostatic induction drives electrons back to the TENG, completing the circuit (iv). The study also investigated how varying loading forces affect the fabric’s ability to generate power, with Voc increasing alongside the applied force. A practical demonstration involved attaching the fabric to a foot sole, where it exhibited measurable power output, highlighting the potential for diverse applications.

Furthermore, conductive fibers can be seamlessly incorporated into conventional fabrics, thus imparting them with circuitry functions. Lee et al. showcased this by integrating conductive fibers with electronic components, such as LEDs or resistors, and sewing them onto fabrics to create wearable circuits, as depicted in Figure 6C. The fibers’ mechanical robustness and suitability for large-scale coating make it feasible to integrate them into commercial fabrics using standard sewing machines, as shown in Figure 6D. Moreover, the successful incorporation of electronic components into large-scale smart garments (Figure 6E) further underscores the reliability of liquid metal conductive fibers, which maintain stable performance even when subjected to mechanical deformation [84]. Collectively, these examples substantiate the versatility and practical utility of liquid metal conductive fibers in the realm of wearable sensors.

## 6. Liquid Metal Coated Fibers 

### 6.1. Preparations

**Figure 7:**
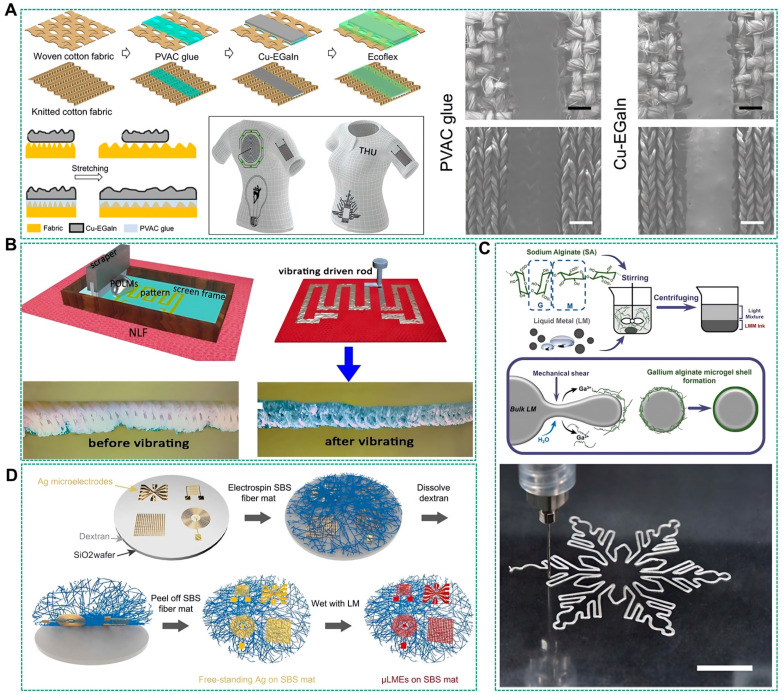
Liquid metal-coated fibers. (**A**) Fabrication of semiliquid-metal-based wearable electronics for smart fabrics and SEM micrographs of woven and knitted cotton fabrics with printed PVAC glue and Cu-EGaIn [95]. Copyright: (2019) American Chemical Society. (**B**) Schematic illustration processes of printing POLMs on the NLF by screen printing and vibrating the POLMs with an air compressor-driven rod [96]. Copyright: (2020) American Chemical Society. (**C**) Schematic diagram of the procedure for preparing the LMM ink with mechanical stirring and images of the process of the direct ink writing patterning on a polyethylene terephthalate film with the LMM ink [97]. Copyright: (2022) American Chemical Society. (**D**) Schematic illustration of the fabrication process of μLMEs [98]. Copyright: (2023) AAAS.

Currently, liquid metal-coated fibers are commonly manufactured by pre-woven fibers to form a fabric. Afterward, the fabric is used as a substrate, and a liquid metal conductive coating is adhered to its surface to create a flexible sensor on the fabric substrate. To achieve stable adhesion of the liquid metal coating on the fabric, materials with high adhesion to liquid metals are used, such as SBS [91] and Ecoflex [92]. Furthermore, to allow high mass loading of liquid metal and possesses a smart conductivity-strain-enhancing feature, the SBS mat was immersed in a precursor solution containing silver trifluoroacetate (Ag+[COOCF3−]), followed by a chemical reduction process with hydrazine to produce the Ag-coated SBS mat. The surface coverage of Ag on the SBS fibers increased proportionally with the concentration of hydrazine. The contact angle of liquid metals with an Ag-coated SBS mat can be reduced significantly, thus enabling the adhesion of more liquid metals [93]. However, fabrics used to manufacture garments are often woven from natural fibers and have a rough surface topography. Previous studies have found that this rough interface makes it difficult for liquid metals to adhere to its surface [94]. Therefore, Guo et al. used screen printing to cover the surface of cotton fabric with a polyvinyl acetate (PVAC) adhesive film. This adhesive film penetrated into the voids between the fabric fibers and formed a smooth and flat interface on the fabric surface. In addition, this adhesive film has excellent adhesion to liquid metals, firmly attaching them to the fabric’s surface, as shown in Figure 7A [95]. Furthermore, to enhance the electrical conductivity and stability of the fabric circuit structure, a semi-liquid metal slurry infused with copper metal microparticles (Cu-EGaIn) was utilized in place of the traditional liquid metal gallium-indium alloy. This Cu-EGaIn slurry, with its higher electrical conductivity and superior molding capabilities, could be rapidly adhered to the PVAC adhesive film using a roller coating technique. To further improve the stability and waterproofing of the liquid metal clothing circuit, an Ecoflex adhesive film was used to encapsulate the liquid metal circuit surface. In another approach, Ou et al. introduced a vibration-assisted printing method for partially oxidized liquid metals (POLMs) to ensure the formation of a stable liquid metal network within nylon lycra fabric (NLF), as shown in Figure 7B [96]. This method first reduces the surface tension of the liquid metals through partial oxidation. The POLMs, susceptible to external forces, are reinforced by vibrating them to fill the inner spaces of cloth fibers, forming a network that is protected by the NLF fiber network, thus maintaining robustness against external forces. As a result, POLMs within the garment exhibit high metallic conductivity and durability without adding extra thickness to the fabric.

In addition to employing oxidized liquid metals to diminish surface tension, a recyclable liquid metal microgel (LMM) ink has been formulated. This ink comprises alginate microgel shells encapsulating liquid metal droplets, as depicted in Figure 7C [97]. Upon mechanical agitation, Ga^3+^ ions released from the LMM can cross-link with sodium alginate, forming a microgel layer that envelops the liquid metal droplets. The ink’s shear-thinning characteristic, attributable to the dynamic formation and rupture of hydrogen bonds under varying stress conditions, endows LMM inks with exceptional printability and robust adhesion to diverse substrates. Although initially non-conductive, the printed patterns can be rendered conductive through activation processes such as microstrain (less than 5%), pressing, and freezing. The LMM inks facilitate direct 3D printing onto fabric substrates, yielding a stable liquid metal coating on fibers, thereby significantly curtailing the production costs of smart fabrics without the need for pre-fabricated templates.

Furthermore, achieving high-precision liquid metal coatings on fabric substrates represents a significant area of research. Zhuang et al. have introduced a strategy for wafer-scale patterning that enables the creation of supersoft, stretchable, and breathable liquid metal microelectrodes (μLMEs) with high resolution [98]. Figure 7D illustrates the fabrication process of these wafer-scale μLMEs, encompassing four principal stages: (i) photolithographic patterning of Ag on a SiO_2_ wafer pre-coated with a thin film of water-soluble dextran; (ii) electrostatic spinning of a fibrous SBS mat over the Ag micropatterns; (iii) dissolution of the dextran layer, enabling the transfer of the Ag micropatterns from the SiO_2_ wafer to the SBS fiber mat; and (iv) selective wetting of the liquid metal on the Ag-coated areas to produce μLMEs. This technique has demonstrated the capability to achieve pattern resolutions down to 2 μm and the formation of ultra-high density μLME arrays, with approximately 75,500 electrodes per cm^2^, on a 4-inch elastomeric fiber felt through photolithography.

### 6.2. Applications

**Figure 8:**
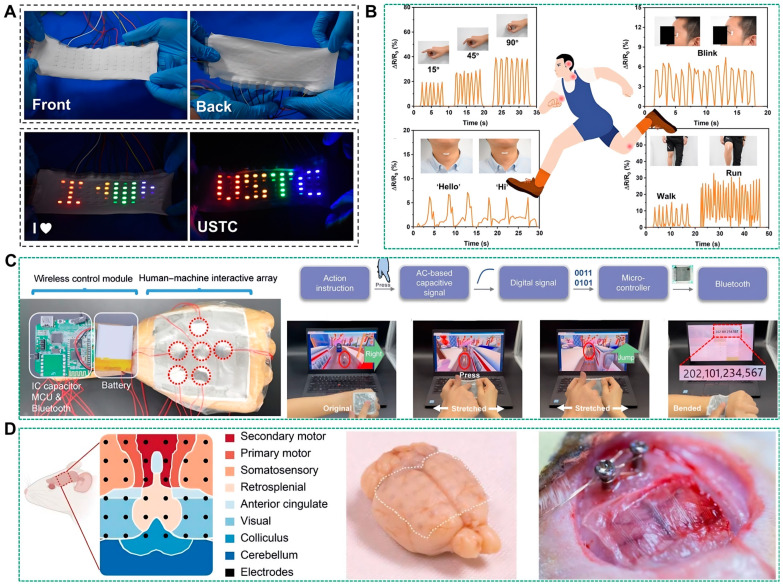
Application of liquid metal-coated fibers. (**A**) Photograph of the front and back of the flexible display [104]. Copyright: (2021) American Chemical Society. (**B**) Monitoring curve of a flexible strain sensor under different bending angles of the finger, small facial expression changes, pronouncing “hello” and “hi,” and subject in walking and running states [104]. Copyright: (2021) American Chemical Society. (**C**) Photographs of the e-skin human-machine interactive array and wireless control module [105]. Copyright: (2022) WILEY. (**D**) Digital images of μLME ECoG electrode array with conformal attachment onto the soft, curved, and sophisticated cerebral cortex [98]. Copyright: (2023) AAAS.

The utilization of liquid metals as conductive coatings on electrospun films represents a prevalent technological approach. Specifically, electrospun films have emerged as optimal substrates for flexible and breathable electronics, attributable to their robust mechanical strength, electrical insulation, ductility, resistance to corrosion, and substantial elasticity [99,100]. However, the high surface tension and fluid nature of liquid metals pose challenges in adhering and patterning on polymer substrates, thereby restricting their application on electrospun substrates [101,102]. Furthermore, existing liquid metal circuits frequently encounter interfacing issues with electronic components such as resistors, capacitors, and inductors [103]. To address these challenges, Wang et al. employed a stencil printing technique to delineate liquid metal patterns onto PU electrospun films [104]. This method, coupled with a layer-by-layer assembly process, facilitates the controllable fabrication of flexible circuits, resistors, capacitors, inductors, and their integrated devices. These devices exhibit enhanced stretchability, permeability, and stability, with the capability to form multilayer structures. The researchers further demonstrated the utility of these devices by designing a 5 × 12 LED display array, depicted in Figure 8A. This display, devoid of rigid internal connections, is microcomputer-controlled and dynamically displays the message “I LOVE USTC”. Additionally, they developed flexible strain sensors for tracking human motion, integrating several of these sensors on a volunteer to evaluate their efficacy in motion monitoring, as illustrated in Figure 8B. These sensors, positioned at finger joints, can detect finger postures while also being sensitive to minute changes in facial expressions. Moreover, the sensors can be affixed to the throat for speech recognition and to the knee for gait analysis. Collectively, these sensors are capable of translating a range of physiological states and motor activities into interpretable, quantifiable, and real-time electrical signals.

Liquid metal circuits integrated with electrospun films offer promising applications in the development of wearable human-machine interface systems, particularly for computer gaming command inputs. For instance, Cao et al. developed a multifunctional electronic skin featuring a two-layer structure [105]. The top layer of this electronic skin is a deformable, adaptive human-machine interactive system, while the bottom layer is an electrothermal heater, both of which exhibit stretchability, conformality, and robustness. This design facilitates a seamless human-computer interface and sustained thermotherapy, as depicted in Figure 8C. The interactive system comprises an array of capacitive sensors capable of transmitting directional commands (up, down, left, right, and pause) when interfaced with a wireless control module. Moreover, the device supports digital input at the wrist, ensuring signal integrity even under positive or negative bending conditions.

Furthermore, Zheng et al. created ultrahigh-density micro-liquid metal electrodes (μLME) arrays for capturing electrocorticography (ECoG) signals from soft, curved, and complex neural interfaces [98]. These μLME arrays were applied to cover major cortical subdomains in rats, including motor, somatosensory, visual, and retrosplenial cortices. The μLME arrays are characterized by small-diameter round electrode units (500 μm), fine interconnections (40 μm), and a high channel density of 100 electrodes per square centimeter. Their mechanical compliance, similar to that of brain tissue, allows for conformal attachment to the cortical surface, as illustrated in Figure 8D. The μLMEs demonstrated long-term biocompatibility, with successful implantation for over 8 months.

## 7. Conclusions

In conclusion, the exploration of liquid metal conductive fibers as a frontier in the realm of biomedical sensing has unveiled a myriad of promising applications and significant advancements. The unique combination of high electrical conductivity, malleability, and biocompatibility of gallium-based liquid metals positions them at the forefront of flexible and wearable biosensing technology. This paper has underscored the versatility of liquid metal-based fibers, highlighting their integration into health monitoring systems, neural interfaces, and wearable devices, which underscore their pivotal role in modern biomedical engineering.

However, the journey towards the widespread adoption of liquid metal conductive fibers is not without its challenges. Issues such as material stability, long-term biocompatibility, and the scalability of production methods require further investigation and resolution. Addressing these concerns will be crucial for the continued evolution and refinement of liquid metal-based biosensors. To solve these issues, one approach is to adjust the composition of liquid metal materials in order to enhance their safety and stability. For example, polymer materials with better biosafety can be modified on the surface of liquid metal. The second is to design a new structure for a liquid metal fiber sensor. For instance, micro-nanostructures can be added to the surface of stretchable tubes or fiber cores to improve the bonding strength and adhesion of liquid metal on their surfaces. Future application directions may include the development of multifunctional fibers that combine sensing, actuation, and data processing capabilities. In addition, the exploration of hybrid materials combining the advantages of liquid metals with other conductive materials may pave the way for the next generation of biosensors with enhanced performance and functionality. Finally, novel fiber processing techniques such as two-photon lithography could be used to fabricate ultrafine liquid metal fiber sensors on the nanoscale for signal detection on the microscale.

## Figures and Tables

**Table 1:** The stretchability and conductivity of conductive materials for fiber sensors.

Materials	Max. Stretchability	Conductivity (S m^−1^)
Au/polydimethylsiloxane	150%	4.5 × 10^7^
Graphene/polyimide	240%	10^6^
Carbon nanotube/polydimethylsiloxane	280%	400
Ag nanoflowers/PU	776%	4.1 × 10^6^
EGaIn	1400%	3.4 × 10^6^
Cu-EGaIn	850%	9.0 × 10^6^

**Table 2:** Summary of the three kinds of liquid metal fibers.

Liquid Material Fiber Types	Characteristics	Fabrication Methods	Applications
Internally filled liquid metal-based tubular textile fiber	High electrical Conductivity High compliance	Injecting	Smart textiles Pressure sensing Energy storage systems
Surface-printed liquid metal-based fiber	Simple manufacturing process Easy to integrate	Coating	Wearable electronics Body electrode Flexible sensors
Liquid metal-coated fiber	Morphological controllability High stretchability	Screen printing 3D printing	Flexible circuits Flexible sensors

## Data Availability

No new data were created or analyzed in this study.
